# Matrix metalloproteinases, tissue inhibitor of metalloproteinase and transforming growth factor A1 in the remodeling of chronic rhinosinusitis in North China

**DOI:** 10.1186/2045-7022-3-S2-O10

**Published:** 2013-07-16

**Authors:** XiangDong Wang, ChengShuo Wang, MingYu Bo, Hong Wang, Ying Li, ErZhong Fan, Nan Zhang, Claus Bachert, Luo Zhang

**Affiliations:** 1 Department of Otorhinolaryngology Head and Neck Surgery of Beijing Tongren Hospital, Beijing Institute, Beijing, China; 2 Beijing Tongren Hospital, Capital Medical University, Department of Otorhinolaryngology, Beijing, China; 3 Beijing Institute of Otorhinolaryngology, Department of Otorhinolaryngology, Beijing, China; 4 Ghent University Hospital, Department of Otorhinolaryngology, Ghent, Belgium; 5 Beijing Tongren Hospital, Capital Medical University, Beijing Institute of Otorhinolaryngology, Department of Otorhinolaryngology, Beijing, China

## Background

To evaluate the expression and roles of matrix metalloproteinases (MMPs), tissue inhibitor of metalloproteinase (TIMP) 1,2,3,4, and transforming growth factor ¦Â1(TGF-¦Â1) in chronic rhinosinusitis without nasal polyps (CRSsNP) and with nasal polyps (CRSwNP) in North China.

## Method

100 cases of NP tissue, 25 cases of sinus mucosa of CRSsNP and 25 cases of control were enrolled in the present study. ELISA was used to measure MMP-1,2,3,7,8,9,12,13 and TGF-¦Â1 in homogenization.

## Results

MMP-8,9 were the highest among all above MMPs. MMP-8 of NP tissue was much higher than that of CRS and control tissue (P<0.001). MMP-9 of NP tissue was also significantly higher than that of CRSsNP and control tissue (P<0.05). MMP-7 of NP tissue and CRSsNP were much higher than that of control tissue (P<0.001). MMP-2 of CRSsNP tissue was much higher than that of NP and control tissue (P<0.001). TIMP-1,2 were the highest among all the four TIMPs. TIMP-1 of CRSsNP tissue was much higher than that of NP and control tissue (P<0.001 and P<0.05 respectively). TIMP-2 of NP and CRSsNP tissue were much higher than that of control tissue (P<0.01). TIMP-3 of CRSsNP tissue was much higher than that of NP and control tissue (P<0.01 and P<0.05 respectively). There were no significantly difference of TGF-¦Â1 among the three groups.

## Conclusion

Different MMPs and TIMPs may play different roles in the remodeling of CRSsNP and CRSwNP.

